# Inverted-Type InAlAs/InAs High-Electron-Mobility Transistor with Liquid Phase Oxidized InAlAs as Gate Insulator

**DOI:** 10.3390/ma14040970

**Published:** 2021-02-18

**Authors:** Yuan-Ming Chen, Hsien-Cheng Lin, Kuan-Wei Lee, Yeong-Her Wang

**Affiliations:** 1 Institute of Electro-Optical Science and Engineering, National Cheng-Kung University, Tainan 701, Taiwan; o6685@hotmail.com; 2 Institute of Microelectronics, Department of Electrical Engineering, National Cheng-Kung University, Tainan 701, Taiwan; pp3129@yahoo.com.tw; 3 Department of Electronic Engineering, I-Shou University, Kaohsiung 840, Taiwan

**Keywords:** inverted-type, InAs, metal-oxide-semiconductor (MOS), high-electron-mobility transistor (HEMT)

## Abstract

An inverted-type InAlAs/InAs metal-oxide-semiconductor high-electron-mobility transistor (MOS-HEMT) with liquid phase oxidized (LPO) InAlAs as the gate insulator is demonstrated. A thin InAs layer is inserted in the sub-channel layers of InGaAs to enhance the device performance. The proposed inverted-type InAlAs/InAs MOS-HEMT exhibits an improved maximum drain current density, higher transconductance, lower leakage current density, suppressed noise figures, and enhanced associated gain compared to the conventional Schottky-gate HEMT. Employing LPO to generate MOS structure improves the surface states and enhances the energy barrier. These results reveal that the proposed inverted-type InAlAs/InAs MOS-HEMT can provide an alternative option for device applications.

## 1. Introduction

The conventional Si-based field effect transistors are reaching the scaling limit in logic devices [1]. High-indium-content compound semiconductors with high electron mobility of more than 8500 cm^2^/Vs, as well as gate insulators, are being considered as alternative channel materials for the future in the complementary metal-oxide-semiconductor field-effect transistor (MOSFET) industry. To achieve low noise figures in future radio frequency (RF) applications, InP-based high-electron-mobility transistors (HEMTs) with InGaAs channels have demonstrated very high levels of performance and have been widely used in circuits operating in the microwave range [2,3]. Many efforts have been made recently to obtain higher performance by designing novel channel structures. For instance, Ng et al. [4] reported that a high indium ratio (65%) InGaAs channel InAlAs/InGaAs HEMT resulted in 13% improvement in terms of mobility (11,200 to 9900 cm^2^/Vs) compared to 53% indium at room temperature. Matsumura et al. [5] reported that inserting an InAs monolayer within the channel of an AlGaAs/InGaAs HEMT resulted in a 15% improvement in mobility compared to a channel without an InAs monolayer at room temperature. Eugster et al. [6] reported that using a thin InAs channel in InAlAs/InAs modulation-doped field-effect transistor (MODFET) resulted in low output conductance and a high breakdown voltage. It is expected that such devices will benefit from the high mobility of InAs, 40,000 cm^2^/Vs, and will thus exhibit high-speed operation and low power consumption. Some studies have also fabricated InAs or InGaAs HEMTs on Si substrates and have shown excellent results [7,8]. In addition, the inverted-type HEMT design [9,10,11] is also an interesting concept (i.e., the carrier supply layer or donor layer is designed below the channel layer). The advantage of an inverted-type HEMT is that the threshold voltage can be easily controlled through gate recess without a reduction in the drain current due to the etching of the upper donor layer. Akazaki et al. [10] reported that the inverted-type HEMT with an InGaAs/InAs/InGaAs channel structure exhibits only a small kink effect and has higher breakdown voltage.

An In_0.52_Al_0.48_As Schottky layer with high aluminum content may suffer from the leakage current issue, which causes a reduction in the breakdown voltage. Additionally, an HEMT with high indium content suffers from impact ionization due to the narrow bandgap channel, which induces a higher gate leakage current. One commonly used method to address this issue is to insert a dielectric layer between the metal gate and the In_0.52_Al_0.48_As layer to supply a high energy barrier. Several laboratories have announced successful results related to the preparation of oxide film on semiconductors, such as thermal oxidation [12], chemical anodization [13], photochemical oxidation [14], molecular beam epitaxy (MBE) [15], and atomic layer deposition (ALD) [16]. In particular, oxidized InAlAs developed using the UV/ozone process [17] and wet oxidation [18] has been reported. Compared with the experimental apparatus mentioned above, the liquid phase oxidation (LPO) [19] operated at near room temperature (e.g., 50 °C) only requires a pH meter and a constant temperature groove to grow an oxide film on semiconductors, whereas a technique that does not require any anodic equipment or assisting energy source is used to grow smooth native oxide films. The technique was successfully demonstrated on InAlAs [20,21,22] and showed promising experimental results for the metamorphic HEMTs on a GaAs substrate with an InGaAs channel. In this work, InAlAs film was oxidized using LPO as the gate insulator to fabricate an inverted-type InAlAs/InAs metal-oxide-semiconductor high-electron-mobility transistor (MOS-HEMT), where a thin InAs layer was inserted within the InGaAs sub-channel layers to improve the device performance.

## 2. Experimental

For preparing the LPO growth solution, gallium-ion-containing nitric acid solution was obtained through the sufficient dissolution of high purity (6N) gallium metal in heat (60 °C) and concentrated nitric acid (70%) for more than 8 h, and was then diluted with deionized (DI) water [19]. Next, the optimum pH value was adjusted by adding an ammonia solution into the nitric acid solution. Finally, a clear solution with a pore size less than 0.1 μm was obtained using filtration. The growth solution was composed of ammonium nitrate salt (NH_4_NO_3_) and a little nitric acid. When the growth solution was heated using a temperature-controlled heater (e.g., 50 °C), the hydrolysis reaction of the ammonium nitrate solution generated ammonia gas and nitric acid. This can be described as Formula (1). Nitric acid oxidizes GaAs-based materials because it is a strong oxidizing reagent. In general, nitric acid is easier to decompose due to absorption heat and can be described as Formula (2).

NH_4_NO_3__(aq)_ → NH_3__(g)_ + HNO_3__(aq)_ (1)

4HNO_3__(aq)_ → 4NO_(g)_ + 3O_2__(g)_ + 2H_2_O_(aq)_ (2)

4In + 3O_2_ → 2In_2_O_3_ (3)

4Al + 3O_2_ → 2Al_2_O_3_ (4)

4As + 3O_2_ → 2As_2_O_3_ (5)

**Figure 1:**
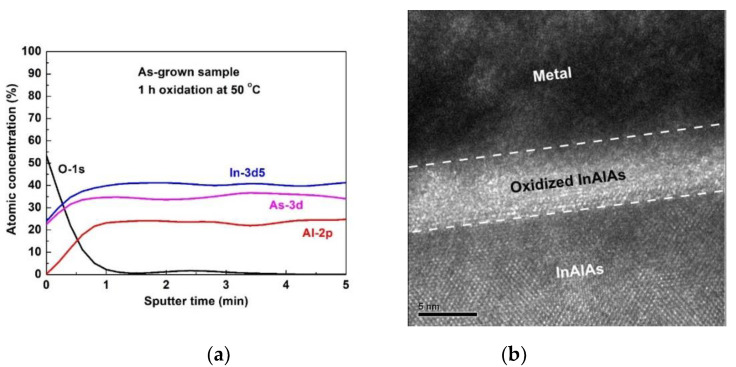
(**a**) XPS depth profiles of the as-grown sample on InAlAs. (**b**) Cross-sectional TEM image of oxidized InAlAs.

Figure 1a demonstrates the X-ray photoelectron spectroscopy (XPS, PHI 5000 VersaProbe, Physical Electronics, Chanhassen, MN, USA) depth profiles of the as-grown sample on InAlAs. The as-grown LPO-oxide film (referring to Formulas (3) to (5)) was composed of Al_2_O_3_, As_2_O_3_, and In_2_O_3_. However, there were almost no Al oxides on the surface after 1 h oxidation, which was similar to the results in reference [20]. Figure 1b shows the cross-sectional transmission electron microscope (TEM, JEOL JEM-2010, Akishima, Japan) image of the oxidized InAlAs. The oxide film on the InAlAs layer was found to be approximately 6 nm thick.

**Figure 2:**
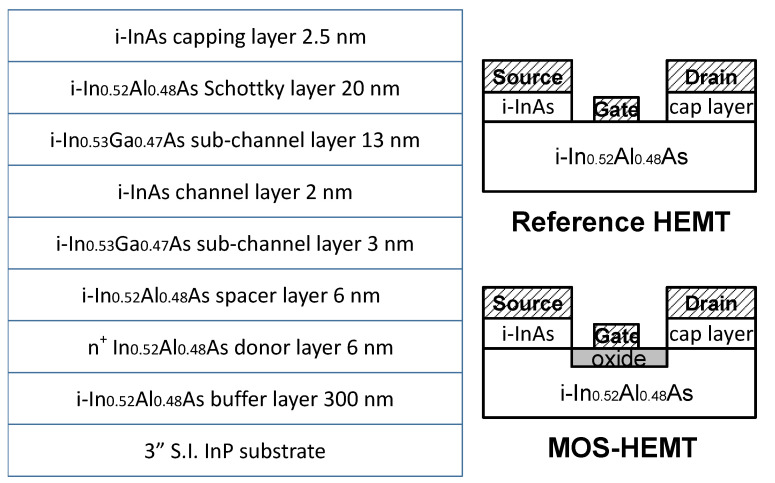
Schematic structures of the Schottky-gate InAlAs/InAs high-electron-mobility transistor (HEMT) and the proposed InAlAs/InAs metal-oxide-semiconductor (MOS)-HEMT.

**Figure 3:**
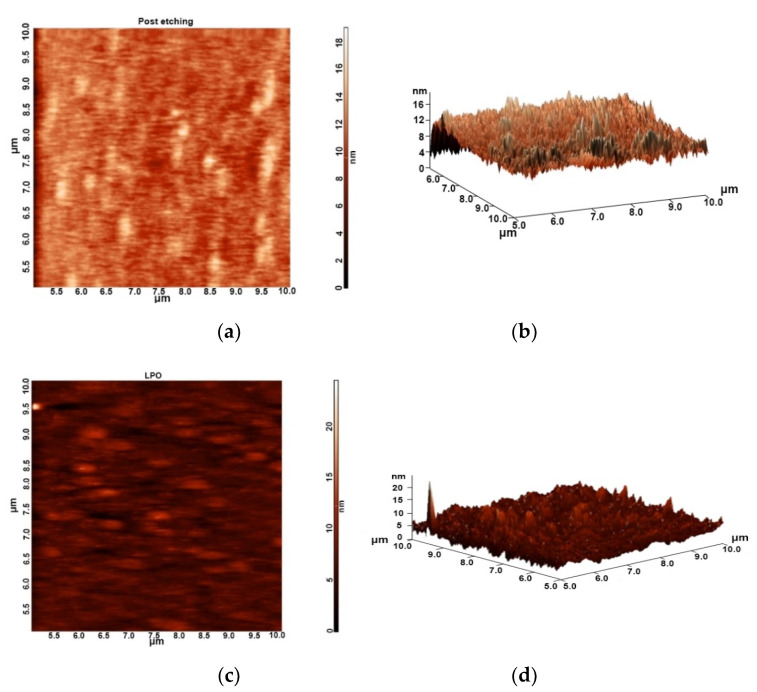
(**a**) 2D and (**b**) 3D atomic force microscopy (AFM) images of post-etching InAlAs layer. (**c**) 2D and (**d**) 3D AFM images of oxidized InAlAs surface.

The device structure was grown using the MBE system on a 3 inch semi-insulating InP substrate. It was composed of a 300 nm undoped InAlAs buffer layer, a 6 nm InAlAs donor layer with a silicon doping density of 4 × 10^18^ cm^−3^, a 6 nm undoped InAlAs spacer layer, a 3 nm undoped In_0.53_Ga_0.47_As sub-channel layer, a 2 nm undoped InAs channel layer, a 13 nm undoped In_0.53_Ga_0.47_As sub-channel layer, a 20 nm undoped InAlAs Schottky layer, and a 2.5 nm undoped InAs capping layer. Room temperature Hall measurements showed electron mobility of 14,262 cm^2^/Vs and a sheet carrier concentration of 2.07 × 10^12^ cm^−2^. Figure 2 shows schematic structures of inverted-type InAlAs/InAs HEMTs as the reference sample and the proposed MOS-HEMTs with oxidized InAlAs as the gate insulator. The device mesa isolation was conducted through a wet etching method using an H_3_PO_4_-based solution. Mesa etching should reach the buffer layer. After forming the ohmic alloy with Au/Ge/Ni by evaporation and annealing at 420 °C for 30 s in an N_2_ atmosphere, gate recess was performed using a citric-acid-based etchant. For MOS-HEMT fabrication, LPO was used to generate the gate insulator by immersing the post-etching samples into the growth solution. The thickness of gate oxide was approximately 11 nm. Finally, the gate metal was deposited with Au for the reference HEMT and the MOS-HEMT. Figure 3a,b shows the 2D and 3D atomic force microscopy (AFM) images of the InAlAs surface after the gate recess process, respectively. The measured area was 5 × 5 μm, and the root mean square (RMS) value of the surface was 1.63 nm. Figure 3c,d shows the 2D and 3D AFM images of the oxidized InAlAs surface after the LPO process, respectively. The related RMS value of the surface was 1.53 nm. The surface roughness of the interface underneath the metal gate influences the DC and RF performance of the HEMTs. Improved surface roughness of oxidized InAlAs using LPO was beneficial to the device performance.

## 3. Results and Discussion

**Figure 4:**
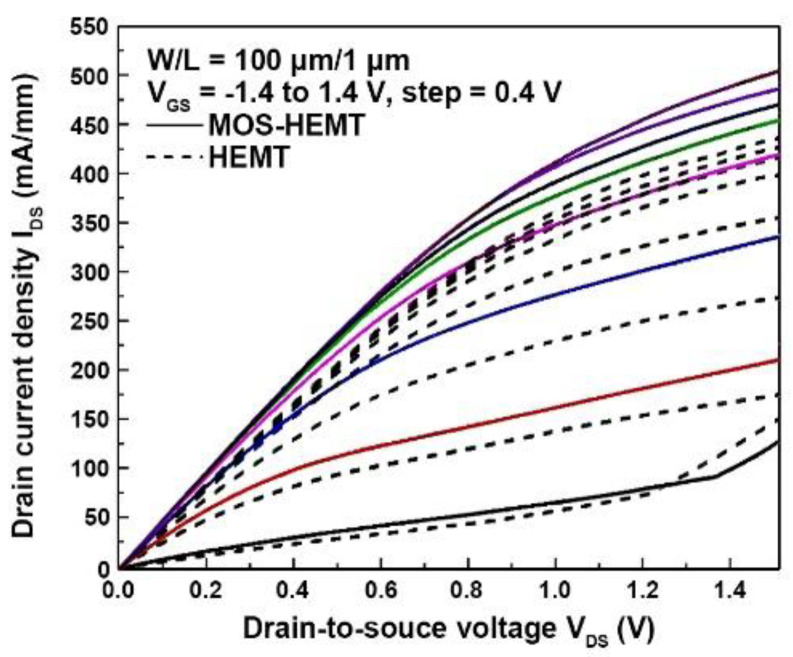
The comparison of measured drain current density (I_DS_) versus the drain-to-source voltage (I_DS_–V_DS_) characteristics for reference HEMT and MOS-HEMT. The oxide thickness is 11 nm for MOS-HEMT.

Figure 4 shows the measured drain current density (I_DS_) versus the drain-to-source voltage (V_DS_) characteristics of the conventional HEMT and MOS-HEMT with a gate length and width of 1 μm and 100 μm, respectively. The gate-to-source voltage (V_GS_) ranged from −1.4 to 1.4 V in 0.4 V steps. The maximum I_DS_ of the reference HEMT was 441 mA/mm at V_DS_ = 1.5 V. The maximum I_DS_ of the MOS-HEMT was 509 mA/mm at V_DS_ = 1.5 V, which is higher than that of the reference HEMT. It was difficult to obtain the completed pinch-off characteristics for both devices. The reason why such devices do not turn off completely has been attributed to the carrier supply layer being far away from the metal gate or donor-type traps located in the lower portion of the energy bandgap at the InAlAs/oxide interface [23], which means the charged state will be positive after losing electrons when negative voltage is applied to the gate.

**Figure 5:**
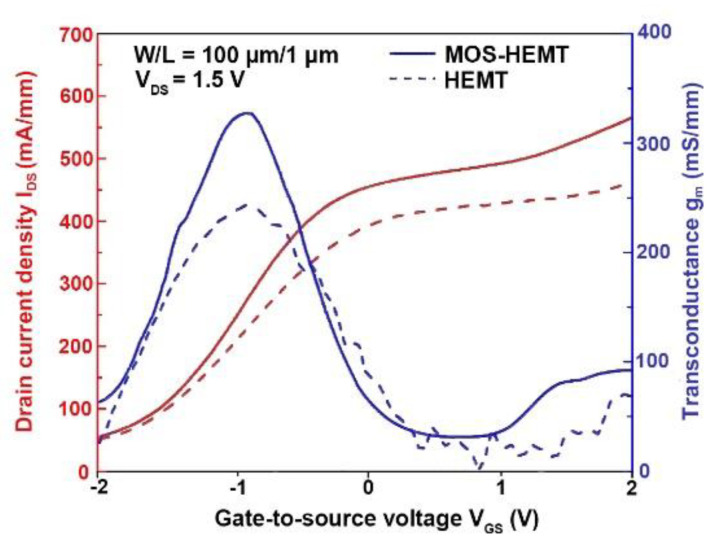
The comparison of transconductance (g_m_) and I_DS_ versus gate-to-source voltage (V_GS_) at a fixed V_DS_ of 1.5 V for the reference HEMT and MOS-HEMT.

The I_DS_ and related transconductance (g_m_) versus V_GS_ at V_DS_ = 1.5 V for both devices are shown in Figure 5. The maximum extrinsic g_m_ was 243 mS/mm and the threshold voltage was −1.9 V for the reference HEMT. The maximum extrinsic g_m_ was 327 mS/mm and the threshold voltage was −1.7 V for the proposed MOS-HEMT. The band bending in the InAlAs Schottky layer is affected at the higher gate bias, and the conduction band has a chance to be lower than the Fermi level, leading to a small g_m_ at higher V_GS_. The enhanced I_DS_ and g_m_ characteristics were attributed to the improvement in the oxide/InAlAs interface.

**Figure 6:**
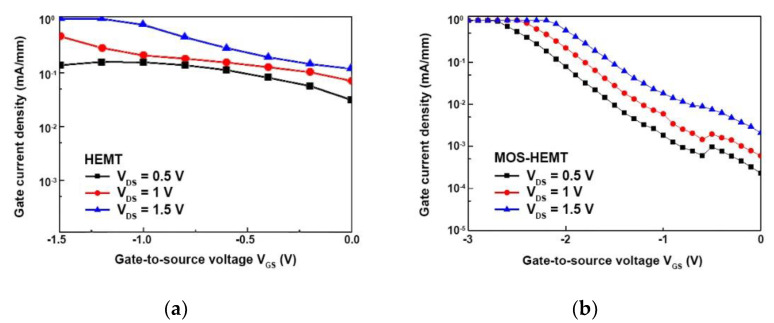
The gate current density versus V_GS_ at distinct V_DS_ for (**a**) reference HEMT and (**b**) MOS-HEMT.

Figure 6a,b demonstrates the measured gate current density as a function of V_GS_ for the reference HEMT and the proposed MOS-HEMT, respectively. The V_DS_ ranged from 0.5 to 1.5 V in 0.5 V steps. The gate current density for the MOS-HEMT was significantly suppressed by at least two orders of magnitude compared to that of the reference HEMT. Please note that this is expressed on a logarithm scale, so the change in the current density is obvious. The enhanced impact ionization was due to the higher electric field existing around the gate-to-drain region where the hot carrier phenomena occurred in the narrow composite channel in the case of the reference HEMT. In the MOS-HEMT, the electric field was weaker than that of the Schottky-gate HEMT owing to the oxidized InAlAs between the metal gate and the InAlAs Schottky layer.

**Figure 7:**
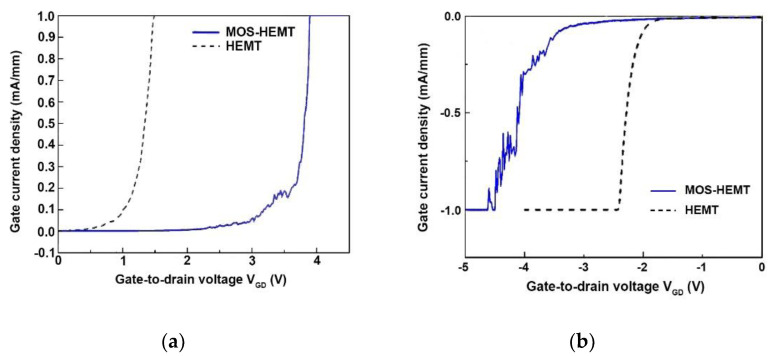
The gate leakage current of two-terminal diode characteristics for (**a**) forward biases and (**b**) reverse biases for both devices.

Figure 7a,b shows the gate leakage current of two-terminal diode characteristics for forward and reverse gate-to-drain voltage (V_GD_) for both devices, respectively. In this measurement, the source terminal was floating and the drain terminal was grounded. The turn-on voltages of the Schottky-gate HEMT and the MOS-HEMT were 1.54 and 3.98 V, respectively. The corresponding reverse gate-to-drain breakdown voltages of the reference HEMT and the MOS-HEMT were −2.5 and −4.6 V, respectively. In general, a higher turn-on voltage or reverse gate-to-drain breakdown voltage accompanies a smaller gate leakage current and higher gate voltage swing. In addition, a larger turn-on voltage allows a higher I_DS_ in the composite channel, which improves the device power and is consistent with the results shown in Figure 5.

**Figure 8:**
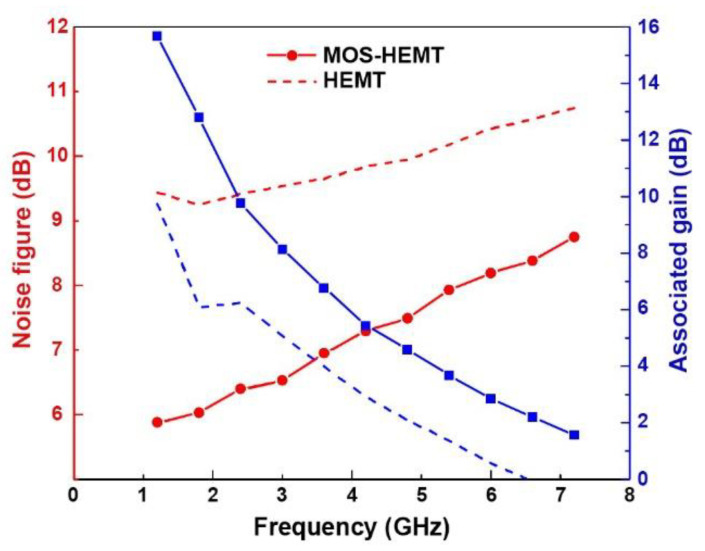
The comparison of noise figure and associated gain for both devices.

With the increasing demands of wireless communication, applications with low noise figures and high associated gains are required [24,25]. Circuit noise is characterized by the noise figure, which is defined as the signal-to-noise ratio at the amplifier output divided by the signal-to-noise ratio at the input. The minimum noise figure (NF_min_) can be obtained as Formula (6):

(6) $$NF_{min}≅1+\left(\frac{f}{f_{T}}\right)K_{f}\sqrt{g_{m}\left(R_{s}+R_{g}\right)}$$

where *f* is the operating frequency; *f*_T_ is cutoff frequency; *K_f_* is the Fukui factor; *Rs* is the source series resistance; *Rg* is the gate series resistance [26]. The formula describes the behavior of GaAs metal-semiconductor field-effect transistor (MESFET) and can be used for a qualitative discussion of high-frequency noise of HEMT. Figure 8 presents a comparison of the noise and gain performances measured at 0.6 GHz intervals between 1.2 and 7.2 GHz for both devices. The measured noise figures of the HEMT (MOS-HEMT) increased from 9.44 dB (5.88 dB) at 1.2 GHz to 10.75 dB (8.75 dB) at 7.2 GHz. The associated gains of the HEMT (MOS-HEMT) decreased from 9.74 dB (15.67 dB) at 1.2 GHz to 0 dB (1.56 dB) at 7.2 GHz. The higher noise figure may have been due to the probe pad resistances during measurement.

The main noise sources of HEMTs include shot noise, hot-electron noise, and generation-recombination noise [27,28]. The Schottky barrier corresponding to the gate current is associated with shot noise. As mentioned previously, owing to the decreased leakage current shown in Figure 6 and Figure 7 for the MOS-HEMT, an improved minimum noise figure was expected. In addition, hot-electron noise due to energetic random electron motion, which can enhance the impact ionization mechanism. Therefore, the reduced impact ionization suppresses the hot-electron noise. Traps at the metal/semiconductor interface may contribute to high-frequency noise [29]. The LPO-grown oxide (i.e., oxidized InAlAs) could decrease dangling bonds to suppress the surface recombination centers to further reduce interface traps. In other words, employing LPO to generate a MOS structure could improve the surface states and enhance the barrier height. In addition, Fermi-level movement is enhanced around the gap as trap density decreases near the interface. However, a very low interface trap density is required for conventional MOSFET, which is different from the composite channel positioned away from the oxidized InAlAs with the Schottky layer and spacer layer for MOS-HEMT. Therefore, the issue of the traps underneath the oxide has little impact on the channel. Based on the above three arguments (i.e., Schottky barrier issue, channel issue, and interface trap issue), the noise figure and associated gains of MOS-HEMT could be improved. The device performances of the inverted-type InAlAs/InAs HEMTs with and without oxidized InAlAs as the gate insulator in this work are summarized in Table 1. Table 2 summarizes the device parameters for the high indium ratio MOS-HEMTs in this work and previous work [20,22].

## 4. Conclusions

In this study, we used the LPO to grow oxidized InAlAs film on a Schottky layer for fabricating inverted-type InAlAs/InAs MOS-HEMT. A thin InAs layer was inserted within the InGaAs sub-channel layer to enhance the device performance. The proposed inverted-type InAlAs/InAs MOS-HEMT exhibited higher drain current density and g_m_ compared with those of the reference HEMT. Besides, a suppressed noise figure and enhanced associated gain can be achieved based on the improved gate current density, decreased impact ionization, and reduced interface traps due to the oxidized InAlAs film. These results indicate that the proposed inverted-type InAlAs/InAs MOS-HEMT can provide an alternative option for device applications.

## Figures and Tables

**Table 1:** Summary of device performances for the inverted-type InAlAs/InAs HEMTs with and without gate oxide in this work.

Gate Oxide	With	Without
Maximum I_DS_ (mA/mm) at V_DS_ = 1.5 V	509	441
Peak g_m_ (mS/mm) at V_DS_ = 1.5 V	327	243
Turn-on voltage (V)	3.98	1.54
Reverse gate-to-drain breakdown voltage (V)	−4.6	−2.5
Minimum noise figure NF_min_ at 1.2 GHz (dB)	5.88	9.44
Associated gain at 1.2 GHz (dB)	15.67	9.74

**Table 2:** Summary of device parameters for proposed oxidized InAlAs on MOS-HEMTs in this work and previous work.

Type	Inverted-Type (This Work)	Normal-Type Ref. [20]	Normal-Type Ref. [22]
Substrate	InP	GaAs	GaAs
Channel	In_0.53_Ga_0.47_As/ InAs/In_0.53_Ga_0.47_As	In_0.53_Ga_0.47_As	In_0.53_Ga_0.47_As
Hall mobility (cm^2^/Vs)/sheet carrier concentration (cm^−2^) @ 300 K	14,262/2.07 × 10^12^	7000/2 × 10^12^	7000/2 × 10^12^
Gate length (μm)	1	0.65	0.65
0.65
Maximum I_DS_ (mA/mm)	509	252	424
Peak g_m_ (mS/mm)	327	226	254
